# 3-[1-(4-Methyl­phen­ylsulfon­yl)-1,4-di­hydro­pyridin-4-yl]-1*H*-indole

**DOI:** 10.1107/S1600536810010214

**Published:** 2010-03-24

**Authors:** Ana M. F. Oliveira-Campos, Joana M. O. Ribeiro, Lígia M. Rodrigues, Pier Parpot, Paulo E. Lopes

**Affiliations:** a Centro de Química, Universidade do Minho, Campus de Gualtar, 4710-452 Braga, Portugal

## Abstract

In the title compound, C_20_H_18_N_2_O_2_S, the indole mean plane and benzene ring form a dihedral angle of 65.0 (1)°. In the crystal structure, weak inter­molecular N—H⋯π and C—H⋯O inter­actions link the mol­ecules into ribbons propagated along [100].

## Related literature

For the pharmacological activity of compounds containing indole and pyridine fragments, see: Fanshawe *et al.* (1970 ▶); Bennasar *et al.* (1990 ▶); Lavilla *et al.* (1997 ▶).
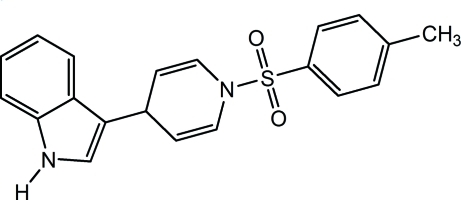


## Experimental

### 

#### Crystal data

C_20_H_18_N_2_O_2_S
*M* *_r_* = 350.42
Orthorhombic, 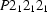

*a* = 7.9192 (9) Å
*b* = 11.4344 (13) Å
*c* = 19.168 (2) Å
*V* = 1735.7 (3) Å^3^
*Z* = 4
Mo *K*α radiation
μ = 0.20 mm^−1^
*T* = 293 K
0.47 × 0.45 × 0.42 mm

#### Data collection

Bruker SMART 1000 CCD diffractometer
11335 measured reflections
4115 independent reflections
2830 reflections with *I* > 2σ(*I*)
*R*_int_ = 0.036

#### Refinement

*R*[*F* ^2^ > 2σ(*F* ^2^)] = 0.041
*wR*(*F* ^2^) = 0.102
*S* = 1.01
4115 reflections
231 parameters
H atoms treated by a mixture of independent and constrained refinement
Δρ_max_ = 0.17 e Å^−3^
Δρ_min_ = −0.24 e Å^−3^
Absolute structure: Flack (1983 ▶), 1718 Friedel pairs
Flack parameter: −0.07 (8)

### 

Data collection: *SMART* (Bruker 2001 ▶); cell refinement: *SAINT* (Bruker 2001 ▶); data reduction: *SAINT*; program(s) used to solve structure: *SHELXS97* (Sheldrick, 2008 ▶); program(s) used to refine structure: *SHELXL97* (Sheldrick, 2008 ▶); molecular graphics: *SHELXTL* (Sheldrick, 2008 ▶); software used to prepare material for publication: *SHELXTL* and *publCIF* (Westrip, 2010 ▶).

## Supplementary Material

Crystal structure: contains datablocks I, global. DOI: 10.1107/S1600536810010214/cv2703sup1.cif

Structure factors: contains datablocks I. DOI: 10.1107/S1600536810010214/cv2703Isup2.hkl

Additional supplementary materials:  crystallographic information; 3D view; checkCIF report

## Figures and Tables

**Table 1:** Hydrogen-bond geometry (Å, °) *Cg* is the centroid of the C1–C6 ring.

*D*—H⋯*A*	*D*—H	H⋯*A*	*D*⋯*A*	*D*—H⋯*A*
C10—H10⋯O1^i^	0.93	2.54	3.370 (3)	149
N1—H1⋯*Cg*^ii^	0.83 (3)	2.51	3.207 (3)	143

Symmetry codes: (i) 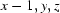; (ii) 
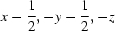
.

**Fig. 1.**
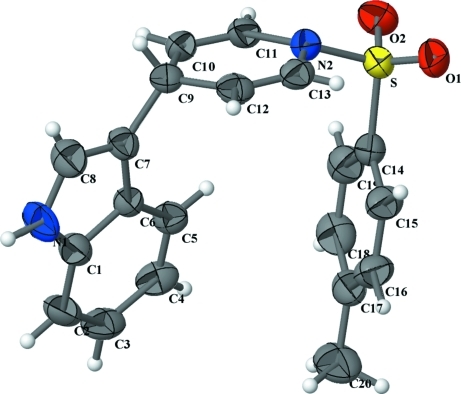
The molecular structure of the title compound showing the atomic numbering and 50 % probability displacement ellipsoids.

## supplementary crystallographic information

### Comment

A vast number of compounds containing both indole and pyridine fragments exhibit pharmacological activity (Fanshawe *et al.* 1970, Lavilla *et al.* 1997). Attempted *N*-tosylation of indole in pyridine gave a product which did not correspond to the expected *N*-tosylindole. Crystallization of this compound from acetonitrile yielded light pink crystals of a compound that by ESI-MS, ^1^H and ^13^C NMR data showed to contain indole, dihydropyridine and the tosyl group. Following studies on the nucleophilic addition of indole to pyridinium salts (Bennasar *et al.*, 1990; Lavilla *et al.* 1997) the structure for the isolated compound was suggested to be 3-{1-[(4-Methylphenyl)sulfonil]-1,4-dihydropyridin-4-yl}-1*H*- indole (Scheme 1) and later confirmed by single-crystal X-ray diffraction (Figure 1).

### Experimental

To a solution of indole (5.0 g; 4.3x10^-2^ mol) in pyridine (10 mL) tosyl chloride (5.4 g; 2.8x10^-2^ mol) was added at 0°C. The mixture was left stirring for 90 minutes, at room temperature, then HCl (10%) was added dropwise until neutral and the mixture was extracted with chloroform (4x20 ml). The organic extracts were combined, dried (MgSO_4_), and evaporated to dryness to give an oily orange solid (3.1 g, η=20%). Crystallization from acetonitrile gave light pink crystals (0.9 g), m.p. 147.8-150.7°C of 3-{1-[(4-Methylphenyl)sulfonil]-1,4-dihydropyridin-4-yl}-1H-indole.

^1^H NMR, (DMSO-d_6_, 300 MHz): δ 10.84 (s, 1H, NH), 7.78 (d, 2H, J = 8.1 Hz, H-2" and H-6"), 7.49 (d, 2H, J = 8.1 Hz, H-3" and H-5"), 7.29 (d, 1H, J=7.8 Hz, H-4), 7.14 (d, 1H, J=7.8 Hz, H-7), 7.01 (t, 1H, J=7.5 Hz, H-6), 6.85 (d, 1H, J=2.4 Hz, H-2), 6.72 (t, 1H, J=7.8, H-5), 6.61 (dd, 2H, J=1,5 e 8.1 Hz, H-2' e H-6'), 5.02 (dd, 2H, J=2.4 e 8.7 Hz, H-3' e H-5'), 4.27 (m, 1H, H-4'), 2.46 (s, 3H, CH_3_). ^13^C NMR (DMSO-d_6_): δ 144.53 (C-4"), 136.60 (C-7a), 134.31 (C-1"), 130.20 (C-3" and C-5"), 126.87 (C-2" and C-6"), 125.78 (C-3a), 122.26 (C-2), 121.06 (C-2' and C-6'), 120.93 (C-6), 118.46 (C-7), 118.21 (C-5 or C-1), 118.16 (C-5 or C-1), 111.71 (C-3' and C-5'), 111.48 (C-4), 29.17 (C-4'), 21.12 (CH_3_). ESI- MS: The molecular ion was not observed, only the ion at 195 (M-155)^+^ corresponding to the loss of tosyl from the molecular ion.

### Refinement

C-bound H atoms  were geometrically positioned (C–H 0.93-0.96 Å) and refined as riding, with *U*_iso_(H) = 1.2-1.5 *U*_eq_(C). Atom H1 was located on a difference map and isotropically refined with bond restraint N–H = 0.85 (3) Å.

### Crystal data

**Table:**

C_20_H_18_N_2_O_2_S	*F*(000) = 736
*M**_r_* = 350.42	*D*_x_ = 1.341 Mg m^−^^3^
Orthorhombic, *P*2_1_2_1_2_1_	Mo *K*α radiation, λ = 0.71073 Å
Hall symbol: P 2ac 2ab	Cell parameters from 2827 reflections
*a* = 7.9192 (9) Å	θ = 2.6–21.5°
*b* = 11.4344 (13) Å	µ = 0.20 mm^−^^1^
*c* = 19.168 (2) Å	*T* = 293 K
*V* = 1735.7 (3) Å^3^	Prism, light pink
*Z* = 4	0.47 × 0.45 × 0.42 mm

### Data collection

**Table:**

Bruker SMART 1000 CCD diffractometer	2830 reflections with *I* > 2σ(*I*)
Radiation source: fine-focus sealed tube	*R*_int_ = 0.036
graphite	θ_max_ = 28.0°, θ_min_ = 2.1°
phi and ω scans	*h* = −10→10
11335 measured reflections	*k* = −10→14
4115 independent reflections	*l* = −25→22

### Refinement

**Table:**

Refinement on *F*^2^	Secondary atom site location: difference Fourier map
Least-squares matrix: full	Hydrogen site location: inferred from neighbouring sites
*R*[*F*^2^ > 2σ(*F*^2^)] = 0.041	H atoms treated by a mixture of independent and constrained refinement
*wR*(*F*^2^) = 0.102	*w* = 1/[σ^2^(*F*_o_^2^) + (0.0423*P*)^2^ + 0.2268*P*] where *P* = (*F*_o_^2^ + 2*F*_c_^2^)/3
*S* = 1.01	(Δ/σ)_max_ < 0.001
4115 reflections	Δρ_max_ = 0.17 e Å^−^^3^
231 parameters	Δρ_min_ = −0.24 e Å^−^^3^
0 restraints	Absolute structure: Flack (1983), 1718 Friedel pairs
Primary atom site location: structure-invariant direct methods	Flack parameter: −0.07 (8)

### Special details

**Table:**

Geometry. All esds (except the esd in the dihedral angle between two l.s. planes) are estimated using the full covariance matrix. The cell esds are taken into account individually in the estimation of esds in distances, angles and torsion angles; correlations between esds in cell parameters are only used when they are defined by crystal symmetry. An approximate (isotropic) treatment of cell esds is used for estimating esds involving l.s. planes.
Refinement. Refinement of *F*^2^ against ALL reflections. The weighted *R*-factor wR and goodness of fit *S* are based on *F*^2^, conventional *R*-factors *R* are based on *F*, with *F* set to zero for negative *F*^2^. The threshold expression of *F*^2^ > σ(*F*^2^) is used only for calculating *R*-factors(gt) etc. and is not relevant to the choice of reflections for refinement. *R*-factors based on *F*^2^ are statistically about twice as large as those based on *F*, and *R*- factors based on ALL data will be even larger.

### Fractional atomic coordinates and isotropic or equivalent isotropic displacement parameters (Å^2^)

**Table:**

	*x*	*y*	*z*	*U*_iso_*/*U*_eq_	
S	0.43816 (8)	0.16555 (6)	0.18936 (3)	0.05332 (19)	
O1	0.5841 (2)	0.12035 (19)	0.22313 (9)	0.0705 (6)	
O2	0.3845 (3)	0.28318 (17)	0.20181 (9)	0.0720 (6)	
N1	−0.1929 (3)	−0.2711 (2)	0.05481 (12)	0.0609 (7)	
H1	−0.249 (4)	−0.330 (3)	0.0446 (15)	0.078 (10)*	
C1	−0.1217 (3)	−0.1920 (2)	0.01063 (12)	0.0458 (6)	
C2	−0.1307 (3)	−0.1842 (3)	−0.06159 (12)	0.0561 (7)	
H2	−0.1872	−0.2404	−0.0877	0.067*	
C3	−0.0535 (4)	−0.0909 (2)	−0.09299 (12)	0.0592 (7)	
H3	−0.0601	−0.0825	−0.1412	0.071*	
C4	0.0354 (3)	−0.0080 (2)	−0.05365 (12)	0.0529 (6)	
H4	0.0885	0.0538	−0.0764	0.063*	
C5	0.0459 (3)	−0.01583 (19)	0.01780 (11)	0.0440 (5)	
H5	0.1060	0.0396	0.0432	0.053*	
C6	−0.0352 (3)	−0.10865 (18)	0.05181 (11)	0.0366 (5)	
C7	−0.0614 (3)	−0.14245 (19)	0.12343 (10)	0.0415 (5)	
C8	−0.1592 (3)	−0.2394 (2)	0.12171 (12)	0.0548 (7)	
H8	−0.1982	−0.2789	0.1609	0.066*	
C9	0.0027 (3)	−0.0848 (2)	0.18946 (11)	0.0444 (6)	
H9	−0.0680	−0.1132	0.2279	0.053*	
C10	−0.0131 (3)	0.0452 (2)	0.18840 (12)	0.0463 (6)	
H10	−0.1194	0.0769	0.1802	0.056*	
C11	0.1134 (3)	0.1181 (2)	0.19833 (11)	0.0459 (6)	
H11	0.0928	0.1979	0.1949	0.055*	
N2	0.2792 (2)	0.08014 (18)	0.21399 (9)	0.0445 (5)	
C13	0.3048 (3)	−0.0418 (2)	0.21675 (11)	0.0473 (6)	
H13	0.4128	−0.0697	0.2260	0.057*	
C12	0.1825 (3)	−0.1175 (2)	0.20673 (10)	0.0462 (6)	
H12	0.2084	−0.1966	0.2105	0.055*	
C14	0.4588 (3)	0.1429 (2)	0.09933 (11)	0.0456 (6)	
C15	0.5422 (3)	0.0451 (2)	0.07507 (12)	0.0542 (6)	
H15	0.5929	−0.0064	0.1063	0.065*	
C16	0.5499 (3)	0.0241 (2)	0.00439 (13)	0.0595 (7)	
H16	0.6064	−0.0419	−0.0117	0.071*	
C17	0.4753 (4)	0.0990 (3)	−0.04315 (12)	0.0563 (7)	
C18	0.3938 (4)	0.1972 (2)	−0.01774 (13)	0.0611 (7)	
H18	0.3451	0.2493	−0.0490	0.073*	
C19	0.3829 (3)	0.2198 (2)	0.05302 (13)	0.0549 (7)	
H19	0.3257	0.2854	0.0692	0.066*	
C20	0.4842 (5)	0.0734 (3)	−0.12010 (14)	0.0867 (11)	
H20A	0.4108	0.1259	−0.1448	0.130*	
H20B	0.4494	−0.0058	−0.1285	0.130*	
H20C	0.5981	0.0838	−0.1361	0.130*	

### Atomic displacement parameters (Å^2^)

**Table:**

	*U*^11^	*U*^22^	*U*^33^	*U*^12^	*U*^13^	*U*^23^
S	0.0401 (3)	0.0752 (5)	0.0447 (3)	−0.0101 (3)	−0.0001 (3)	−0.0173 (3)
O1	0.0388 (10)	0.1244 (18)	0.0481 (9)	−0.0081 (11)	−0.0087 (8)	−0.0082 (10)
O2	0.0725 (13)	0.0654 (12)	0.0783 (13)	−0.0150 (10)	0.0113 (10)	−0.0355 (10)
N1	0.0710 (17)	0.0531 (14)	0.0587 (14)	−0.0285 (13)	−0.0069 (11)	−0.0050 (12)
C1	0.0458 (13)	0.0460 (14)	0.0457 (12)	0.0012 (12)	−0.0048 (10)	−0.0067 (11)
C2	0.0607 (15)	0.0638 (18)	0.0438 (13)	0.0057 (14)	−0.0102 (12)	−0.0176 (13)
C3	0.0673 (18)	0.0776 (19)	0.0328 (12)	0.0201 (17)	−0.0006 (13)	0.0021 (12)
C4	0.0640 (17)	0.0497 (14)	0.0451 (13)	0.0098 (13)	0.0084 (12)	0.0085 (12)
C5	0.0489 (14)	0.0384 (12)	0.0446 (12)	−0.0006 (12)	0.0027 (11)	−0.0016 (10)
C6	0.0359 (13)	0.0353 (11)	0.0384 (11)	0.0033 (10)	−0.0015 (9)	−0.0017 (9)
C7	0.0428 (12)	0.0451 (13)	0.0366 (11)	−0.0043 (11)	−0.0039 (10)	0.0020 (9)
C8	0.0609 (17)	0.0559 (17)	0.0475 (14)	−0.0164 (13)	−0.0008 (12)	0.0081 (12)
C9	0.0428 (12)	0.0585 (15)	0.0318 (11)	−0.0053 (11)	0.0023 (10)	−0.0024 (11)
C10	0.0342 (11)	0.0581 (15)	0.0465 (12)	0.0069 (11)	−0.0015 (10)	−0.0145 (12)
C11	0.0410 (12)	0.0518 (14)	0.0450 (13)	0.0097 (11)	−0.0029 (10)	−0.0137 (11)
N2	0.0351 (10)	0.0594 (14)	0.0391 (10)	0.0019 (9)	−0.0016 (8)	−0.0063 (9)
C13	0.0421 (13)	0.0651 (18)	0.0346 (11)	0.0115 (13)	−0.0017 (10)	−0.0013 (11)
C12	0.0553 (15)	0.0525 (15)	0.0309 (11)	0.0059 (13)	−0.0031 (10)	0.0011 (10)
C14	0.0373 (13)	0.0566 (15)	0.0429 (12)	−0.0058 (12)	0.0018 (10)	−0.0064 (11)
C15	0.0490 (15)	0.0706 (18)	0.0429 (13)	0.0124 (14)	−0.0001 (12)	0.0012 (12)
C16	0.0561 (16)	0.0716 (18)	0.0509 (14)	0.0103 (15)	0.0084 (13)	−0.0096 (13)
C17	0.0550 (17)	0.0714 (18)	0.0424 (13)	−0.0195 (14)	0.0036 (12)	0.0032 (12)
C18	0.0675 (18)	0.0613 (18)	0.0544 (15)	−0.0117 (15)	−0.0071 (13)	0.0167 (14)
C19	0.0552 (16)	0.0484 (14)	0.0611 (16)	−0.0029 (13)	0.0023 (13)	−0.0008 (13)
C20	0.103 (3)	0.114 (3)	0.0430 (15)	−0.029 (2)	0.0031 (16)	0.0028 (16)

### Geometric parameters (Å, °)

**Table:**

S—O1	1.4217 (19)	C15—C16	1.377 (3)
S—O2	1.431 (2)	C16—C17	1.383 (4)
S—N2	1.662 (2)	C17—C18	1.383 (4)
S—C14	1.753 (2)	C17—C20	1.505 (4)
N1—C8	1.359 (3)	C18—C19	1.383 (3)
N1—C1	1.361 (3)	N1—H1	0.83 (3)
C1—C2	1.389 (3)	C2—H2	0.93
C1—C6	1.415 (3)	H3—C3	0.93
C2—C3	1.369 (4)	C4—H4	0.93
C3—C4	1.401 (4)	C5—H5	0.93
C4—C5	1.375 (3)	C8—H8	0.93
C5—C6	1.401 (3)	H9—C9	0.98
C6—C7	1.441 (3)	C10—H10	0.93
C7—C8	1.353 (3)	C11—H11	0.93
C7—C9	1.514 (3)	C12—H12	0.93
C9—C10	1.492 (3)	C13—H13	0.93
C9—C12	1.509 (3)	C15—H15	0.93
C10—C11	1.316 (3)	C16—H16	0.93
C11—N2	1.415 (3)	C18—H18	0.93
N2—C13	1.410 (3)	C19—H19	0.93
C13—C12	1.314 (3)	C20—H20A	0.96
C14—C15	1.379 (3)	C20—H20B	0.96
C14—C19	1.386 (3)	C20—H20C	0.96
			
O1—S—O2	120.47 (12)	C18—C17—C20	121.6 (3)
O1—S—N2	105.83 (11)	C19—C18—C17	121.7 (3)
O2—S—N2	106.28 (11)	C18—C19—C14	118.8 (2)
O1—S—C14	108.58 (11)	C1—N1—H1	128 (2)
O2—S—C14	109.32 (12)	C8—N1—H1	123 (2)
N2—S—C14	105.29 (10)	N1—C8—H8	124.6
C8—N1—C1	109.2 (2)	C7—C8—H8	124.7
N1—C1—C2	130.0 (2)	C1—C2—H2	121.1
N1—C1—C6	107.5 (2)	C3—C2—H2	121.1
C2—C1—C6	122.5 (2)	C2—C3—H3	119.5
C3—C2—C1	117.8 (2)	C4—C3—H3	119.5
C2—C3—C4	121.0 (2)	C3—C4—H4	119.2
C5—C4—C3	121.5 (2)	C5—C4—H4	119.3
C4—C5—C6	119.0 (2)	C4—C5—H5	120.5
C5—C6—C1	118.2 (2)	C6—C5—H5	120.5
C5—C6—C7	135.4 (2)	C7—C9—H9	107
C1—C6—C7	106.32 (19)	C12—C9—H9	107
C8—C7—C6	106.19 (19)	C10—C9—H9	107
C8—C7—C9	124.7 (2)	C9—C10—H10	117.8
C6—C7—C9	129.1 (2)	C11—C10—H10	117.8
C7—C8—N1	110.7 (2)	C10—C11—H11	118.5
C10—C9—C12	109.2 (2)	N2—C11—H11	118.6
C10—C9—C7	113.21 (19)	C9—C12—H12	117.8
C12—C9—C7	113.0 (2)	C13—C12—H12	117.8
C11—C10—C9	124.4 (2)	C12—C13—H13	118.7
C10—C11—N2	122.9 (2)	N2—C13—H13	118.6
C13—N2—C11	116.4 (2)	C14—C15—H15	120.2
C13—N2—S	118.87 (16)	C16—C15—H15	120.2
C11—N2—S	117.57 (17)	C15—C16—H16	119.3
C12—C13—N2	122.7 (2)	C17—C16—H16	119.3
C13—C12—C9	124.4 (2)	C17—C18—H18	119.1
C15—C14—C19	120.4 (2)	C19—C18—H18	119.1
C15—C14—S	119.76 (18)	C18—C19—H19	120.5
C19—C14—S	119.75 (19)	C14—C19—H19	120.6
C16—C15—C14	119.6 (2)	C17—C20—H20A	109.5
C15—C16—C17	121.4 (2)	C17—C20—H20C	109.4
C16—C17—C18	118.0 (2)	C17—C20—H20B	109.5
C16—C17—C20	120.4 (3)	H20A—C20—H20C	109.5

### Hydrogen-bond geometry (Å, °)

**Table:**

Cg is the centroid of the C1–C6 ring.

**Table:**

*D*—H···*A*	*D*—H	H···*A*	*D*···*A*	*D*—H···*A*
C10—H10···O1^i^	0.93	2.54	3.370 (3)	149
N1—H1···Cg^ii^	0.83 (3)	2.51	3.207 (3)	143

Symmetry codes: (i) *x*−1, *y*, *z*; (ii) *x*−1/2, −*y*−1/2, −*z*.
